# COVID-19 outbreak in a psychiatric hospital: what makes it worse?

**DOI:** 10.1186/s12991-022-00403-4

**Published:** 2022-07-11

**Authors:** Marouan Zoghbi, Chadia Haddad, Wael Khansa, Elie Karam, Angela Chamoun, Dory Hachem

**Affiliations:** 1 Department of Psychiatry, Psychiatric Hospital of the Cross, P.O. Box 60096, Jal El dib, Lebanon; 2 Family Medicine, Saint Joseph University, Beirut, Lebanon; 3 INSPECT-LB (Institut National de Santé Publique, d’Épidémiologie Clinique et de Toxicologie-Liban), Beirut, Lebanon; 4 School of Health Sciences, Modern University for Business and Science, Beirut, Lebanon; 5 Faculty of Medicine and Medical Sciences, Holy Spirit University of Kaslik (USEK), Jounieh, Lebanon; 6 Faculty of Sciences, Lebanese University, Fanar, Lebanon

**Keywords:** COVID-19, Mental disorders, Psychiatric hospitals, Outbreak, Symptoms, COVID-19 illness severity

## Abstract

**Background:**

Psychiatric patients could be at risk of worse outcomes from COVID-19 than the general population. The primary objective of the present study was to describe the symptoms and clinical characteristics of COVID-19 patients living in long-term hospital for mental illness in Lebanon. The secondary objective was to evaluate the factors related to COVID-19 disease severity among these patients.

**Methods:**

A retrospective observational study was conducted from September 2020 to January 2021 at the Psychiatric Hospital of the Cross. The total number of COVID‐19 patients in the infected floors is 410 out of 548. The outcome variable was the severity of COVID-19 illness classified into five categories: asymptomatic, mild, moderate, severe and critically ill.

**Results:**

The rate of infection in the affected floors was 74.81%. Almost half of the patients were asymptomatic (49.3%), 43.4% had hyperthermia and only 28.0% had tachycardia and 25.1% developed hypoxia. The multivariate regression analysis showed that higher temperature (ORa = 6.52), lower saturation (ORa = 0.88), higher BMI (ORa = 1.12), higher CRP (ORa = 1.01), being a female (ORa = 4.59), having diabetes (ORa = 8.11) or COPD (ORa = 10.03) were significantly associated with the increase of the COVID-19 severity.

**Conclusions:**

The current study showed that a high rate of infection from COVID-19 was detected in a psychiatric hospital with the majority having asymptomatic to mild symptoms. Female psychiatric patients, desaturation, increase inflammation and comorbidities such as diabetes and COPD were associated with the severity of COVID‐19 among psychiatric patients. Future studies are needed to better understand the causal relation of the factors with severity and long term effects or sequelae of the disease.

## Introduction

Psychiatric patients is one of the most vulnerable populations affected by the COVID-19 pandemic [1–3].This vulnerability is related to higher transmission and poor outcomes. Most individuals with severe mental illness live in institutional environment, such as hospital, units for treatment, hostels, nursing home, and prisons [4, 5]. These populations are at increased risk of infectious disease outbreaks as they reside in close overcrowded environment and interact closely and frequently with nursing staff, and use shared spaces, such as bathrooms and dining room [6]. In addition, psychiatric patients has high rates of physical health comorbidities, such as obesity, hypertension, and metabolic syndrome, as well as high rates of neurocognitive impairment [7–10]. They are more likely than the general population to become infected with COVID-19, and they are more than twice as likely to develop a severe outcome of COVID-19 infection [11, 12].

Risk factors among psychiatric patients for severe infection are highly prevalent, such as increased rates of obesity, cardiovascular disease and chronic obstructive pulmonary disease [8, 13–15]. In addition, gender is a risk factor for increased severity and death in individuals with COVID-19. Previous coronavirus outbreaks, such as SARS-CoV-2 (2002) and the Middle Eastern respiratory syndrome coronavirus (MERS, 2012), revealed gender inequalities in their expression, with males typically being more seriously afflicted than women [16–18]. In COVID-19 infection some studies found a greater incidence of infection in males [19–22], whereas others found no difference in COVID-19 infection rates between men and women [23–26]. This might be due to a higher risk of exposure in males, as well as, access to healthcare and diagnostic options [26]. In addition, the lifestyle factors, including smoking and physical inactivity could have an effect on the disease prognosis [13]. Also, treatment with antipsychotics could be associated with COVID-19 infection severity [13].

In Lebanon, the Psychiatric hospital of the Cross (HPC), the largest inpatient psychiatric hospital, had responded rapidly to the COVID-19 outbreak in the country by adopting confinement measures such as temporarily stopping new inpatient admissions and prohibiting visits from family members to existing inmates. The outpatients’ department was also closed and the medical students were turned to online sessions. Despite these measures, two outbreaks of COVID-19 among patients were observed in the HPC in September 2020 and January 2021. In response to this outbreak, the infected wards were fully isolated. All patients in the ward as well the staff were tested for COVID-19 and those who turned positive were immediately shifted to isolation. Adequate staff training was launched and personal protection equipment was properly supplied. Another unit was created inside the hospital premises to take care of patients needing oxygen supplementation and any form of non-invasive ventilation. Patients requiring invasive ventilation were transferred to COVID ICU floors in other hospitals in a medically assisted transport ambulance.

Early diagnosis and identification of COVID-19 infection in psychiatric patients and prompt response in terms of infection control measures improve treatment outcomes [27]. As far as we know, data scarcity had reported an outbreak in psychiatric settings. In China, an outbreak occurred in a hospital, where 50 inpatients and 30 health professionals were tested positive in February 2020 [4]. In USA, 63 psychiatry hospitals had outbreaks of COVID-19 and as an example one patient became symptomatic and tested positive for COVID-19; almost 80% of the other patients who were living in the same building (*n* = 65) were tested positive within 2 weeks [28, 29]. The scarcity of information is known about the prognostic factors in COVID-19 management in patients with severe mental diseases and living in chronic care facilities. The primary objective of the present study was to describe the symptoms and clinical characteristics of COVID-19 patients living in long-term hospital for mental illness in Lebanon. The secondary objective was to evaluate the factors related to COVID-19 disease severity among these patients.

## Methods

### Study design and subjects

A retrospective cross-sectional study was conducted during two local outbreaks of COVID-19 between September 2020 and January 2021 at the Psychiatric Hospital of the Cross. The inclusion criteria were patients with chronic mental illness treated as internal patients at the aforementioned hospital and with a confirmed diagnosis of COVID-19. During the first outbreak, 94 male inpatients were identified as having COVID-19 and in the second outbreak in January 2021, 316 patients were identified as having COVID-19. The total sample of detected COVID-19 patients were 410.

### Procedures and assessments

Data was extracted from medical files and nursing notes of included patients. Nursing note in the hospital are written by the nursing staff and every info is checked by a second nurse to ensure data validity and avoid errors. Non-additional data was required for the study outside the routine data collected. Daily notes were reviewed when necessary.

The data collected were clinical data that include: socio-demographic characteristics (age, gender and Body mass index (BMI)), the daily COVD-19 symptoms (cough, throat, diarrhea, conjunctivitis, headache, loss of smell, difficulty breathing, chest pain), vital signs (body temperature, pulse rate, respiration rate, SpO2 and blood pressure), laboratory tests (counts of white blood cells and red blood cells, neutrophils, and lymphocytes, liver enzymes and C-reactive protein (CRP)), comorbidities (diabetes, coronary heart disease, hypertension, being tested for tuberculosis (purified protein derivative (PPD) test) and chronic obstructive pulmonary disease (COPD)). In addition, the treatment (Psychiatric and medical treatment) was collected, such as antipsychotic medications, mood stabilizing medications, benzodiazepines, anti-epileptic, antidepressants, anti-hypertensive, anti-diabetic, anti-coagulant and statin medications. The BMI was calculated as body weight in kilograms divided by the square of the height in meters (kg/m2).

The severity of COVID-19 illness was considered as the outcome variable and defined according to the WHO guidelines [3] and the COVID-19 treatment guidelines issued by the National Institutes of health (NIH) [30]. Patients were classified into five categories according to the following criteria: Asymptomatic: Individuals who are tested positive for SARS-CoV-2 using a virologic test (i.e., a nucleic acid amplification test or an antigen test) but have no symptoms. Mild illness: Individuals who have any of the various signs and symptoms of COVID-19 (e.g., fever, cough, sore throat, malaise, headache, muscle pain, nausea, vomiting, diarrhea, loss of taste and smell) but do not have shortness of breath or dyspnea or hypoxia. Moderate illness: Individuals who show evidence of lower respiratory disease during clinical assessment and who have saturation of oxygen (SpO2) ≥ 94%. Severe illness: Individuals who have oxygen saturation < 94%, a ratio of arterial partial pressure of oxygen to fraction of inspired oxygen (PaO2/FiO2) < 300 mm Hg or a respiratory frequency > 30 breaths/min. Critical illness: Individuals who have respiratory failure, septic shock, and/or multiple organ dysfunction.

### Statistical analysis

Data were analyzed on SPSS software version 25. A descriptive analysis was performed using absolute frequencies and percentages for categorical variables, means and standard deviations (SD) for quantitative measures. For testing group differences with the outcome variable, Chi-square test or Fisher’s exact test was used for categorical variables and ANOVA F tests were used for the continuous variable.

Ordinal logistic regression was conducted taking the COVID-19 illness categories as the dependent variables. All the variables that showed a *p* value < 0.2 in the bivariate analysis were included in the model to eliminate potential confounding factors. A *P* value less than 0.05 was considered significant.

## Results

### Sociodemographic and COVID-19 symptoms characteristics

The mean age of included patients was 52.42 ± 16.63 years, with 66.3% males. Half of the patients were smokers 51.2% and the mean BMI was 23.73 ± 3.30. The rate of infection in the affected floors was 74.81%. Almost half of the patients were asymptomatic (49.3%), 43.4% had hyperthermia (43.4%), only 28.0% had tachycardia and 25.1% had low saturation. The reported mortality rate from COVID-19 diagnosis up to 27 days of follow-up was 4.6% (Table 1). Among 149 psychiatric women residing in the hospital, 140 (93.9%) have been infected with COVID-19; however, 60.3% of psychiatric men got the virus.

**Table 1:** Description of COVID-19 symptoms among psychiatric patients (*n* = 410)

	Frequency
COVID-19 illness classification	
Asymptomatic	202 (49.3%)
Mild	145 (35.4%)
Moderate	5 (1.2%)
Severe	37 (9.0%)
Critical	21 (5.1%)
COVID-19 symptoms	
Cough	36 (8.8%)
Throat pain	5 (1.2%)
Diarrhea	12 (2.9%)
Conjunctivitis	8 (2.0%)
Headache	23 (5.6%)
Loss of smell	5 (1.2%)
Difficulty breathing	17 (4.1%)
Chest pain	8 (2.0%)
Temperature	
Normal temperature	232 (56.6%)
Hyperthermia	178 (43.4%)
Pulse	
Normal	295 (72.0%)
Tachycardia	115 (28.0%)
Saturation	
Normal	307 (74.9%)
Abnormal	103 (25.1%)
Patients requiring oxygen supplementation in the psychiatric institution	30 (7.3%)
Patients requiring mechanical ventilation	4 (1.0%)
Patients transferred to specialized hospital	18 (4.4%)
Death	19 (4.6%)

### Description of comorbidities

The prevalence of diabetes among our sample was 7.8%, hypertension (12.0%), coronary heart disease (2.7%) and COPD comorbid association rate was 3.9%. Only 23.4% have a positive reaction to the PPD test (Table 2).

**Table 2:** Description of the comorbidities among psychiatric patients

	Frequency (%)
Diabetes	
Yes	32 (7.8%)
No	378 (92.2%)
Hypertension	
Yes	49 (12.0%)
No	361 (88.0%)
Coronary heart disease	
Yes	11 (2.7%)
No	399 (97.3%)
Chronic obstructive pulmonary disease	
Yes	16 (3.9%)
No	394 (96.1%)
Reaction to the PPD test	
Positive reaction	96 (23.4%)
Negative reaction	314 (76.6%)

### Description of the medications

The majority of the patients were taking typical antipsychotics (73.4%) and only 32.4% were taking the atypical one. More than half of the patients were taking anti-cholinergic (64.3%), 45.6% were taking anti-epileptic and 36.3% were taking benzodiazepines. Almost half of the patients were taking more than one neuroleptic (49.5%). Other treatments taken by the patients are detailed in Table 3.

**Table 3:** Description of the treatment used by the psychiatric patients

	Frequency (%)
Psychiatric treatment	
Atypical ATP	
Yes	133 (32.4%)
No	277 (67.6%)
Typical ATP	
Yes	301 (73.4%)
No	109 (26.6%)
Mood stabilizer	
Yes	37 (9.0%)
No	373 (91.0%)
Benzodiazepines	
Yes	149 (36.3%)
No	261 (63.7%)
Antiepileptic	
Yes	187 (45.6%)
No	223 (54.4%)
Anticholinergic	
Yes	54 (64.3%)
No	30 (35.7%)
Antidepressant SSRI	
Yes	21 (5.1%)
No	389 (94.9%)
Antidepressant TCA	
Yes	18 (4.4%)
No	392 (95.6%)
Number of neuroleptics used	
0	67 (16.3%)
1	140 (34.1%)
> 1	203 (49.5%)
Medical treatment	
Anti-hypertensive	
Yes	69 (16.8%)
No	341 (83.2%)
Anti-diabetic	
Yes	46 (11.2%)
No	364 (88.8%)
Anti-coagulant	
Yes	23 (5.6%)
No	387 (94.4%)
Statin	
Yes	31 (7.6%)
No	379 (92.4%)

### Bivariate analysis

The bivariate analysis taking the COVID-19 illness categories as the dependent variable is presented in Table 4. The results showed that a significantly higher proportion of female patients have severe to critical cases as compared to male patients. Those having diabetes and COPD had more severe critical cases compared to those who did not have these diseases. A higher proportion of patients who take anti-epileptic medication and antipsychotic treatment had less critical cases as compared to those who do not take these medications. Those who take Benzodiazepines had more severe cases as compared to those who do not take this medication. In addition, a higher mean age, temperature, pulse and higher CRP level were significantly associated with severe to critical cases of COVID-19. However, a lower mean saturation and lower mean number of typical antipsychotic were significantly associated with critical cases of COVID-19.

**Table 4:** Bivariate analysis taking the COVID-19 illness categories as the dependent variable

	Asymptomatic	Mild	Moderate	Severe	Critical	*p* value
	Frequency (%)	Frequency (%)	Frequency (%)	Frequency (%)	Frequency (%)	
Gender						
Male	126 (46.3%)	113 (41.5%)	5 (1.8%)	15 (5.5%)	13 (4.8%)	** < 0.001**
Female	76 (55.1%)	32 (23.2%)	0 (0%)	22 (15.9%)	8 (5.8%)	
Diabetes						
Yes	10 (31.3%)	13 (40.6%)	1 (3.1%)	7 (21.9%)	1 (3.1%)	**0.037**
No	192 (50.8%)	132 (34.9%)	4 (1.1%)	30 (7.9%)	20 (5.3%)	
Hypertension						
Yes	22 (44.9%)	18 (36.7%)	0 (0.0%)	9 (18.4%)	0 (0.0%)	0.057
No	180 (49.9%)	127 (35.2%)	3 (1.4%)	28 (7.8%)	21 (5.8%)	
Coronary heart disease						
Yes	8 (72.7%)	0 (0.0%)	0 (0.0%)	1 (9.1%)	2 (18.2%)	0.057
No	194 (48.6%)	145 (36.6%)	5 (1.3%)	36 (9.0%)	19 (4.8%)	
Chronic obstructive pulmonary disease						
Yes	1 (6.3%)	8 (50.0%)	1 (6.3%)	3 (18.8%)	3 (18.8%)	**0.001**
No	201 (51.0%)	137 (34.8%)	4 (1.0%)	34 (8.6%)	18 (4.6%)	
Smoking						
Yes	98 (46.7%)	88 (41.9%)	2 (1.0%)	19 (9.0%)	3 (1.4%)	**0.002**
No	104 (52.0%)	57 (28.5%)	3 (1.5%)	18 (9.0%)	18 (9.0%)	
Atypical ATP						
Yes	72 (54.1%)	44 (33.1%)	1 (0.8%)	14 (10.5%)	2 (1.5%)	0.130
No	130 (46.9%)	101 (36.5%)	4 (1.4%)	23 (8.3%)	19 (6.9%)	
Typical ATP						
Yes	154 (51.2%)	115 (38.2%)	5 (1.7%)	25 (8.3%)	2 (0.7%)	** < 0.001**
No	48 (44.0%)	30 (27.5%)	0 (0%)	12 (11.0%)	19 (17.4%)	
Anti-epileptic						
Yes	85 (45.5%)	76 (40.6%)	3 (1.6%)	19 (10.2%)	4 (2.1%)	**0.031**
No	117 (52.5%)	69 (30.9%)	2 (0.9%)	18 (8.1%)	17 (7.6%)	
Benzodiazepine						
Yes	76 (51.0%)	57 (38.3%)	0 (0%)	16 (10.7%)	0 (0%)	**0.003**
No	126 (48.3%)	88 (33.7%)	5 (1.9%)	21 (8.0%)	21 (8.0%)	

	Mean ± SD	Mean ± SD	Mean ± SD	Mean ± SD	Mean ± SD	
Age	48.78 ± 17.10	47.92 ± 17.72	64.66 ± 2.08	63.50 ± 10.60	60.63 ± 11.91	**0.002**
BMI	23.14 ± 3.09	24.08 ± 3.25	26.03 ± 5.15	24.13 ± 3.56	26.72 ± 2.43	0.097
Temperature	36.89 ± 0.34	38.36 ± 0.79	38.46 ± 0.64	39.48 ± 1.03	39.70 ± 0.88	** < 0.001**
Pulse	91.80 ± 12.20	96.24 ± 11.83	103.66 ± 13.86	90.77 ± 10.64	102.85 ± 19.29	**0.001**
Saturation	97.11 ± 2.46	95.55 ± 3.40	89.00 ± 3.00	89.45 ± 6.87	88.25 ± 3.77	** < 0.001**
Number of typical antipsychotic	1.16 ± 0.87	1.30 ± 0.95	1.40 ± 0.54	1.10 ± 0.96	0.95 ± 0.30	** < 0.001**
Number of atypical antipsychotic	0.39 ± 0.55	0.31 ± 0.49	0.20 ± 0.44	0.45 ± 0.64	0.09 ± 0.30	0.078
CRP	13.80 ± 22.82	33.05 ± 40.27	86.20 ± 98.89	80.36 ± 58.31	75.89 ± 61.33	** < 0.001**

Values marked in bold are significant

### Multivariable analysis

**Fig. 1:**
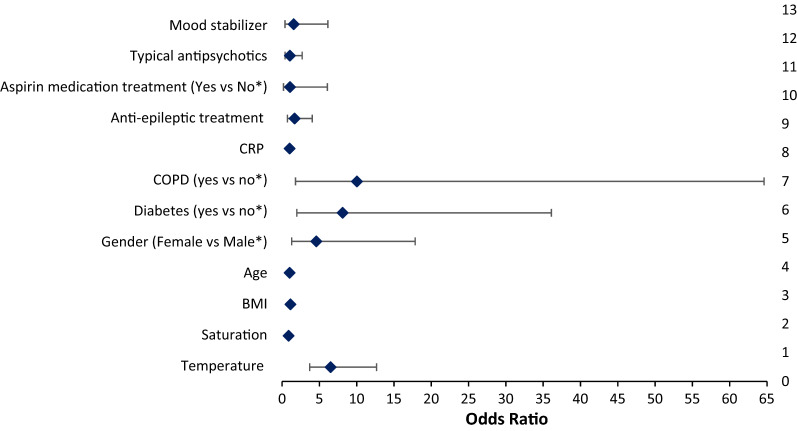
Factors associated with the COVID-19 illness among psychiatric patients

The ordinal regression analysis, taking the severity of the COVID-19 disease as the dependent variable, showed that higher temperature (ORa = 6.52), lower saturation (ORa = 0.88), higher BMI (ORa = 1.12), higher CRP (ORa = 1.01), being a female (ORa = 4.59), having diabetes (ORa = 8.11) and COPD (ORa = 10.03) were significantly associated with the increase on the COVID-19 severity. The use of treatment (anti-epileptic, antipsychotic, and mood stabilizer) were not associated with the severity of the COVID-19 (p > 0.05) (Table 5). The two most contributor factors to the severity of the COVID-19 were COPD and diabetes followed by gender and temperature (Fig. 1).

**Table 5:** Ordinal regression analysis taking the COVID-19 illness categories as the dependent variable

	Beta	*p* value	ORa	95% Confidence interval	
				Lower bound	Upper bound
Temperature	1.874	** < 0.001**	6.520	3.704	12.672
Saturation	-0.124	**0.016**	0.883	0.794	0.972
BMI	0.118	**0.016**	1.125	1.025	1.244
Age	0.015	0.360	1.016	0.982	1.051
Gender (Female vs Male*)	1.524	**0.022**	4.591	1.281	17.834
Diabetes (yes vs no*)	2.093	**0.004**	8.110	1.980	36.095
COPD (yes vs no*)	2.306	**0.011**	10.039	1.805	64.611
CRP	0.011	**0.018**	1.012	1.002	1.022
Anti-epileptic treatment (Yes vs No*)	0.523	0.232	1.689	0.718	4.044
Aspirin medication treatment (Yes vs No*)	0.074	0.933	1.077	0.189	6.076
Typical antipsychotics (Yes vs No*)	0.040	0.933	1.041	0.405	2.694
Mood stabilizer (Yes vs No*)	0.441	0.522	1.556	0.400	6.130

*Reference group

**Values marked in bold are significant.**

## Discussion

The main result of the study is a female, having diabetes and COPD, higher BMI, higher temperature and desaturation is related to a higher COVID-19 severity. However, we could not find any statistically significant association after adjustment between the medication used and the severity of COVID-19. COVID-19 pandemic has hit multiple parts of the HPC hospital in many ways as it is more considered as residential institution, where an overcrowded number of patients with different psychiatric disease live together. In this hospital, 74.81% of the patients from the infected floors were infected by COVID-19. These numbers come in alignment with the rates in some psychiatric wards like the building 0 in a US state psychiatric hospital setting, where the rate of infection was around 78% (51 out of 65 patient) [29]. Given the big number of patients in the hospital, the physical structure of psychiatric units, the difficulties faced in isolating patients with behavioral problems and the limited number of nurses needed to monitor patients have raised the number of COVID-19 infection. In addition, several patients were unable to abide by social distancing rules because of their cognitive and psychiatric conditions. Therefore, the rapid infection in the psychiatric ward was high whenever one patient or a health care worker was tested positive.

When comparing the rates of symptomatic infection among psychiatric inpatients with the general population of Lebanon, psychiatric patient had a lower risk of developing a severe/critical infection (14% vs 20% in the general population) [31]. This could be explained by the closer monitoring of patients and early interventions done. Another explanation could be related to a protective effect of some neuroleptics as suggested by Gordon et al. [32], the anti-viral properties of haloperidol and chlorpromazine, and the typical antipsychotic widely used in the hospital could be a potential explanation for this decreased risk. The low rate of severe cases raises the possible positive effect of those drugs and their anti-viral properties.

On another level, ivermectin was used by the majority of patients (71.2%) as part of treatment at the discretion of the treating physician and this may have reduced the progressing numbers to severe disease.

In our study, 49.3% of patients were tested positive while staying asymptomatic. These numbers match to a certain degree with the previous study done in building 0, where 88% of asymptomatic patients were tested positive for COVID-19 and the rate of asymptomatic infected patients was around 30% of all positive cases in building 0 [29]. It is also notable that our study shows 33% of positive patients having only mild symptoms in line with the numbers found in building 0, where around 25% of positive patients had only mild symptoms [29]. These numbers show that the clear predominance of asymptomatic and mildly symptomatic patients between positive cases.

The regression analysis of our study shows that higher temperature and desaturation is related to a higher COVID-19 severity. This comes in line with the meta-analysis done by Li et al. [33], where both fever and dyspnea were associated with an increased severity of the COVID-19 infection [33]. A retrospective study done in China among eighty COVID-19 patients have demonstrated that the COVID-19 severity was associated with fever and PO2 < 80 mm Hg on admission [34]. Our results suggest that dyspnea and fever are risk factors of severe illness, therefore, requiring a closer monitoring of these patients for earlier intervention.

In our study, having diabetes and COPD were associated with a higher risk of severe infection, in line with the meta-analysis done by Jia li et al., patients having any comorbidity, including COPD and diabetes had a higher rate of severe illness. This suggests that any comorbidity should be assessed in positive patients for closer monitoring of their illness.

It is also important to note that our study found that a higher CRP was associated with an increased risk of severe infection in line with Jia li et al. meta-analysis in which CRP level was also associated with an increased risk of severe illness and ICU admission [33]. Therefore, a close monitoring of the CRP levels could be helpful in predicting severe illness especially when it comes to mass testing in hospitals with a big number of patients. By that minimizing the blood work up for a rapid evaluation of the severity of the COVID-19 infections.

In our study a higher BMI was also associated with a higher COVID-19 severity like with a systemic review and a meta-analysis of 22 studies done by Yanan Chu et al., where obesity and increased BMI were associated with severe illness and worst outcomes [35]. Given that psychiatric patients are at higher risk of obesity given the well-known side effects of SGA, this suggests that long term monitoring of weight is important on the long run, especially during a pandemic like COVID-19.

The results of this study showed that females have higher risk to develop severe COVID-19 illness than men. The number of studies that particularly assess the sex differences in COVID-19-related symptoms was limited, and the results were conflicting. The study done by Takahashi et al. have found that men were at a higher risk of developing a severe illness [36]. In addition, prior research had suggested that male patients had a greater severity and fatality rate [37–39]. A study done by Wang et al. found that women with a recent diagnosis of a mental disorder had higher odds of COVID-19 infection than men after adjusting for age, ethnicity and medical comorbidities [11]. The results could be explained by the higher infection rate among women as reported by an internal hospital report that showed that the incidence of the virus among women were 93.9% and among men was 60.3% that could explain partially the results. In addition, other risk variables might be related to the severity of the disease among women, such as age and the number of medical comorbidities. Moreover, it might be the presence of biological difference in receptors expression (angiotensin-converting enzyme-2 and transmembrane protease serine 2) between males and females as well as immunological differences [40, 41]. Females' have lower production of pro-inflammatory interleukin-6 (IL-6) following viral infection that could explain the development of COVID-19 symptoms [40, 41]. More studies are needed to better understand the association between the gender and the severity of COVID-19 among psychiatric patients.

Another aspect in studying the severity of infection among psychiatric patient was the effect of medications, in particular psychotropic. In the bivariate analysis the use of typical antipsychotics and anti-epileptic by the patients were associated with less COVID-19 severity. However, this association was not found in the multivariate analysis. The use of psychotropic medication and their effect on COVID-19 severity is still debated in the literature as several studies have showed that COVID-19-related severe complications were found to be more common in individuals with psychotic disorders [12, 42]. A recent retrospective study conducted by Canal-Rivero et al. among 698 patients that found that psychiatric patients using antipsychotics and adhering to therapy were less likely to catch COVID-19 and had better outcomes after infection than the general population [43]. The latter study showed that antipsychotic drugs could have a protective effect against COVID-19 lowering the risk of contracting the virus or may have milder symptoms if they do get the virus [43]. A case–control study done by Mckeigue et al. [44] showed polypharmacy’s association with an increased risk of severe infection and the highest risk associated with antipsychotics and drugs with anticholinergic effects [44]. Another study done by Bilbul M. et al. (2020) showed that there is no strict contraindication to the use of psychotropic medication [45]. There are growing evidence that antipsychotics have anti-inflammatory characteristics that may attenuate the normal defensive function of the immune system and possibly reduce uncontrolled inflammatory responses like those found in severe COVID-19 [46]. As for the anti-epileptic drug certain medications may affect the immune system and may increase the risk of infectious diseases 

(47). The consultation–liaison psychiatrist should assist the medical team for using the best psychotropic medication balanced with medical treatment to better manage psychiatric patients infected with COVID-19.

## Limitation

The type of the study was cross sectional that could not infer causality between the associated factors. Second, the results could not be generalized to the whole psychiatric population as the patients were selected from one single site. Due to the limited sample size, comorbidities and certain signs and symptoms did not show any significant association with the severity of the COVID-19 disease. Moreover, age was not one of the most predictive factors related to severity in this study as most of the patients have the same age range around 55 years. In addition, the basic sociodemographic characteristics were not included in this study. The patients' information and severity of the disease might be biased as the information was collected from the medical file and some of them were relied on the nurse memory that might have been affected by recall bias. A selection biased could also occur, since some patients were considered as positive COVID-19 cases without any laboratory confirmation test. The treatment collection and the patients’ characteristics were collected after the COVID-19 period which could prevent the detection of a direct effect of treatment and some important factors on the severity of the disease. Residual confounding bias is also possible, since some factors related to the severity of the COVID-19 were not assessed in this study.

## Conclusions

The current study showed that a high rate of infection from COVID-19 was detected in a psychiatric hospital. However, the majority of psychiatric patients showed asymptomatic and mild symptoms and have lower risk of developing a severe/critical infection. Female psychiatric patients, desaturation, increase inflammation and comorbidities such as diabetes and COPD were associated with the severity of COVID‐19 among psychiatric patients. Strict management of crowded floors in chronic psychiatric institutions remains the cornerstone of treatment and taking additional measures like universal masking before even having the first positive case remains the golden intervention. The identification of risk factors may be helpful for early surveillance of COVID-19 disease progression among psychiatric patients living in psychiatric institution. Clinicians should be aware of the neuropsychiatric side effects of the drugs used to treat COVID-19 patients. Some adjustments may be required to existing psychotropic or the avoidance of specific drugs when receiving medical drugs for COVID-19. More studies are needed to better understand the effect of psychotropic use and causal relation of some factors for managing mental disorders among patient with COVID-19.

## Data Availability

The data sets used and/or analyzed during the current study are available from the corresponding author on reasonable request.

## Abbreviations

COVID-19: Coronavirus disease 2019
SARS-CoV-2: Severe acute respiratory syndrome coronavirus 2
WHO: World Health Organization
HPC: Psychiatric hospital of the Cross
ICU: Intensive care unit
USA: United State of America
PCR: Polymerase chain reaction
BMI: Body mass index
SpO2: Peripheral oxygen saturation
CRP: C-reactive protein
PPD: Purified protein derivative
COPD: Chronic obstructive pulmonary disease
NIH: National Institutes of health
PaO2/FiO2: Pressure of Arterial Oxygen to Fractional Inspired Oxygen Concentration
SPSS: Statistical Package for the Social Sciences
SD: Standard deviations
ATP: Antipsychotic
SSRI: Selective serotonin reuptake inhibitors
TCA: Tricyclic antidepressants
ORa: Adjusted Odds Ratio
SGA: Second-generation antipsychotics
FDA: Food and Drug Administration
MERS: Middle Eastern respiratory syndrome coronavirus
